# Supplementary material for: Direct, Indirect, and Self-Trapped Excitons in Cs2AgBiBr6
Source: J Phys Chem Lett. 2024 Aug 13;15(33):8549–54. doi: 10.1021/acs.jpclett.4c01604 (PMC11345835; doi:10.1021/acs.jpclett.4c01604)
Supplement: Supplementary file 1 — jz4c01604_si_001.pdf [file jz4c01604_si_001.pdf]

Supporting Information:

Direct, Indirect, and Self-Trapped Excitons in $Cs_2AgBiBr_6$

Mehmet Baskurt[1], Paul Erhart[1], and Julia Wiktor[1,*]

[1] *Department of Physics, Chalmers University of Technology, SE-41296, Gothenburg, Sweden*
[*] *julia.wiktor@chalmers.se*

1 Computational details

All calculations in the main text are performed using the Projector Augmented-Wave (PAW) method, as implemented in the VASP software package [1, 2]. The atomic positions in the unit cell were relaxed using the SCAN+rVV10 functional [3], which has been shown to provide very good predictions of the structural properties of halide perovskites [4]. The valence states considered are $5s^25p^66s^1$ for Cs, $4d^{10}5s^1$ for Ag, $6s^26p^3$ for Bi, and $4s^24p^5$ for Br. In a recent study Wang *et al.* [5] showed that including semicore states has a significant effect on the band gap of $Cs_2AgBiBr_6$ when combining norm-conserving pseudopotentials and hybrid functionals. We verified that, when using the PAW method, including one additional shell among the valence states of Ag, Bi, and Br increases the band gap by only 0.08 eV and 0.05 eV with the DDH and PBE0(28%) functionals, respectively. Therefore, we conclude that the issue observed by Wang *et al.* is specific to norm-conserving pseudopotentials.

2 Band structure of $Cs_2AgBiBr_6$

$Cs_2AgBiBr_6$ is an indirect band gap semiconductor with the valence band maximum at the X point and the conduction band minimum at the L point. We plot the band structure of the material in Figure 1.

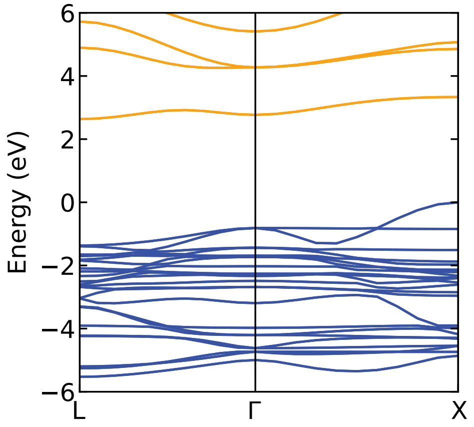

Figure 1: Band structure of $Cs_2AgBiBr_6$ calculated within the PBE0(28%) functional.

3 Convergence tests

We calculate the frequency-dependent dielectric function of $Cs_2AgBiBr_6$ in the unit cell within the Random Phase Approximation (RPA). We verify the convergence with the cutoff energy and the k-

point grid and find that the dielectric function is well converged with the grid of 4×4×4 and cutoff of 400 eV.

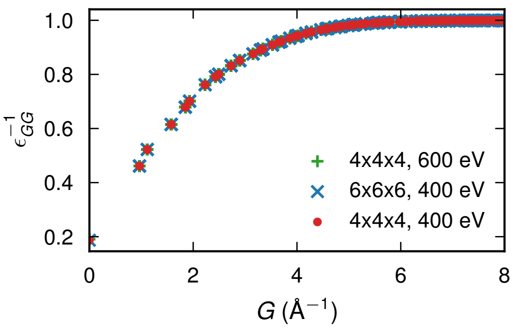

Figure 2: Convergence of the dielectric function with respect to the k-point grid and cutoff energy.

We evaluate the convergence of the absorption spectrum by calculating the imaginary part of the dielectric function within the TD-PBE0(28%) method in the unit cell with varying parameters. We find that the lower part of the spectrum is well converged using the cutoff of 200 eV, the k-point grid of 6×6×6 and considering 10 occupied and 10 unoccupied bands in the Casida equation. We note that all calculation include the Tamm-Dancoff approximation.

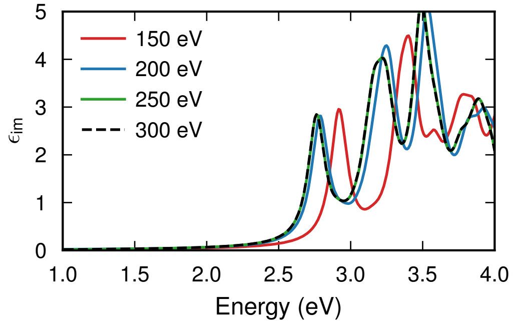

Figure 3: Convergence of the imaginary part of the dielectric function with respect to the cutoff energy.

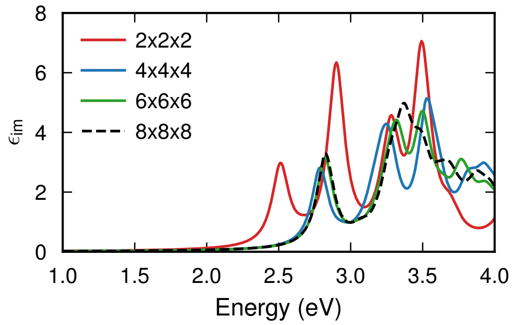

Figure 4: Convergence of the imaginary part of the dielectric function with respect to the k-point grid.

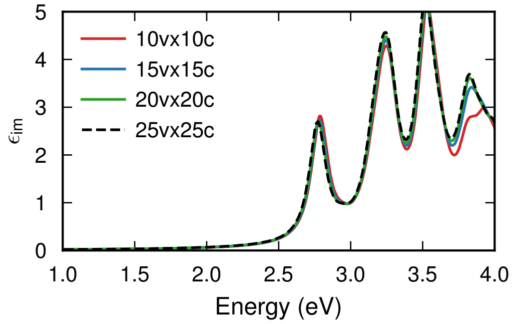

Figure 5: Convergence of the imaginary part of the dielectric function with respect to numbers of occupied and unoccupied bands included in the Casida equation.

We perform separate convergence tests for the supercell. While the cutoff energy is not affected by the cell size, we verify the convergence of the imaginary part of the dielectric function with respect to the k-point mesh and the number of occupied and unoccupied states in the Casida equation. To speed up the convergence with the number of k-points, we consider a special grid with 4 points at (0,0,0), (0,0.5,0.5), (0.5,0,0.5), and (0.5,0.5,0). This grid gives a dielectric function in good agreement with the $2\times2\times2$ grid and is used in the main text. We observe that increasing the density of k-points shifts the peaks in the imaginary part of the dielectric function to slightly higher energies. Conversely, increasing the number of states considered in the Casida equation shifts the peaks to lower energies. Therefore, we anticipate a significant cancellation of errors between these two convergence parameters. In the main text, we present the values calculated with the 4-point k-point grid and with 175 valence and conduction states.

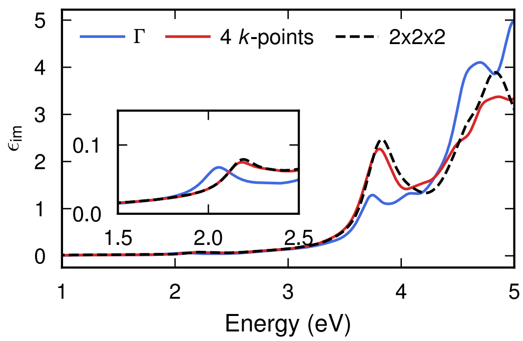

Figure 6: Convergence of the imaginary part of the dielectric function calculated in the geometry of the STE (on the ground state) with respect to the number of k-points. SOC effects have been excluded in this test.

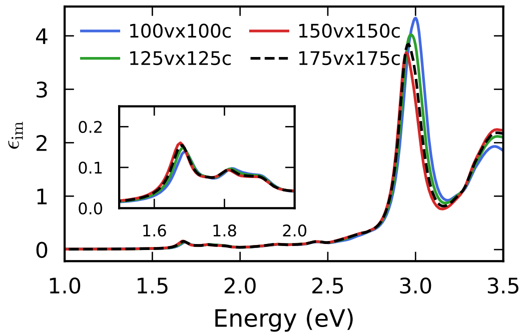

Figure 7: Convergence of the imaginary part of the dielectric function calculated in the geometry of the STE (on the ground state) with respect to the number of occupied and unoccupied bands included in the Casida equation. SOC effects are included in this test.

4 Effect of the fraction of exact exchange on the exciton binding energy

We find the exciton binding energy (E_b) of the first bright exciton (calculated with respect to the fundamental direct band gap) to amount to 550 and 420 meV in in TD-PBE0(28%) and TD-DDH, respectively, as calculated within the unit cell. These values are higher than the previous computational estimates of for example 170 meV in Ref. [6] and 340 meV in Ref. [7]. At the same time, it has been shown that the exciton binding energy is correlated with the band gap [6] and we here calculate larger band gap than in most previous studies. To demonstrate this relationship within our computational setup, we recalculate the exciton binding energies with the TD-PBE0(α) method, where we vary the amount of exact exchange α. Table S1 shows the dependence of E_b on α and the fundamental direct band gap E_{gap}^{dir}. We observe that with the smaller values of α the band gap is closer to what was calculated in Refs. [6] and [7], and so is the exciton binding energy.

Table S1: Exciton binding energy as a function of α and the band gap (E_{gap}^{dir}) in the TD-PBE0(α) calculations.

α	E_{gap}^{dir} (eV)	E_b (meV)
10%	2.34	90
20%	2.90	290
28%	3.36	550

5 Absorption properties in the polymorphous supercell vs. unit cell

In the main text, we benchmark the computational setups by comparing the calculated absorption spectrum with the experimental data. These calculations are performed in the F$m\bar{3}m$ unit cell. The properties of the STE are then studied in a polymorphous supercell. We here test how the absorption of the pristine material compares within the polymorphous supercell and the unit cell. The comparison is given in Figure 8. We observe that when the atomic positions are relaxed with the same method (here SCAN+rVV10), the lower-energy parts of the dielectric function match well between the unit cell and the polymorphus cubic cell. When atomic positions in the polymorphous supercell are relaxed within PBE0(28%), the first peak is shifted by about 0.14 eV to higher energies. In the analysis of the energetics of the excited states in $Cs_2AgBiBr_6$ we consider calculations in which atomic position are relaxed with the PBE0(28%) method, as this is required to obtain the correct charge localization within the STE.

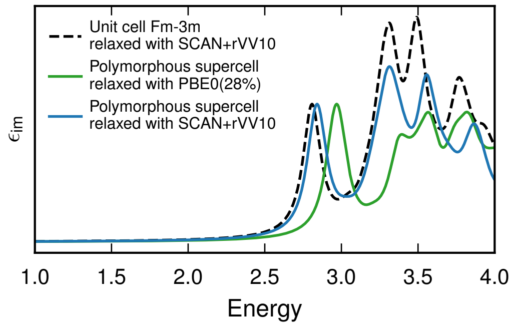

Figure 8: Comparison between the imaginary part of the dielectric function calculated within the F$m\bar{3}m$ unit cell (atomic positions relaxed with SCAN+rVV10) and the cubic supercell (atomic positions relaxed with PBE0(28%) and SCAN+rVV10, respectively). The dielectric functions include a convolution with Lorentzians with a width of 0.07 eV and were normalized to have the same intensity at the first peak.

6 STE in polymorphous vs. tetragonal cell

$Cs_2AgBiBr_6$ has, on average, a cubic structure at room temperature. However, halide perovskites in the cubic phase have been shown to have significant local octahedral tilts, resembling lower temperature structures [4, 8, 9]. For this reason, we have used the polymorphous model of the cubic structure to study the STE. Nevertheless, it is useful to test if the low-temperature tetragonal structure leads to similar properties of the STE, as it displays similar local tilts as the dynamical cubic structure. Therefore, we performed additional TD-PBE0(28%) calculations on a tetragonal supercell. The test has been done using only the Γ point. The comparison between the transitions achieved in the polymorphous and tetragonal supercells is given in Figure 9.

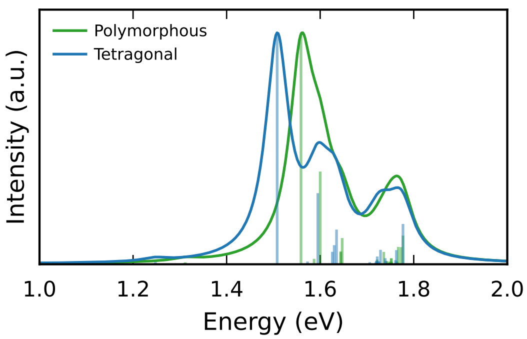

Figure 9: Transitions related to the STE in the polymorphous and tetragonal cells. The broadened spectra were generated by convolution with Lorentzians with a width of 0.03 eV. Calculations were performed considering the Γ point only.

7 STE emission in PBE0(28%) vs. DDH

We test the effect of the choice of the hybrid functional on the transitions related to the STE. In Figure 10 we compare the results achieved with the TD-PBE0(28%) and TD-DDH methods. Using TD-PBE0(28%), the transitions are found at energies lower by about 0.14 eV than in TD-DDH, consistent with the differences in the absorption spectra in the pristine material found within these two methods.

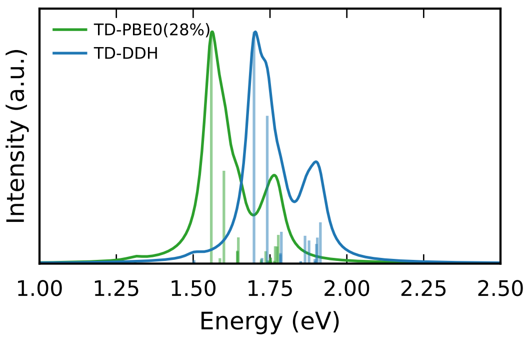

Figure 10: Transitions related to the STE calculated within the TD-PBE0(28%) and TD-DDH methods The broadened spectra were generated by convolution with Lorentzians with a width of 0.03 eV. Calculations were performed considering the Γ point only.

References

[1] Georg Kresse and Jürgen Hafner. Ab initio molecular dynamics for liquid metals. *Phys. Rev. B*, 47(1):558, 1993.

[2] Georg Kresse and Jürgen Furthmüller. Efficient iterative schemes for ab initio total-energy calculations using a plane-wave basis set. *Phys. Rev. B*, 54(16):11169, 1996.

[3] Haowei Peng, Zeng-Hui Yang, John P Perdew, and Jianwei Sun. Versatile van der Waals density functional based on a meta-generalized gradient approximation. *Phys. Rev. X*, 6(4):041005, 2016.

[4] Julia Wiktor, Erik Fransson, Dominik Kubicki, and Paul Erhart. Quantifying dynamic tilting in halide perovskites: Chemical trends and local correlations. *Chem. Mater.*, 35(17):6737–6744, 2023.

[5] Haiyuan Wang, Runhai Ouyang, Wei Chen, and Alfredo Pasquarello. High-quality data enabling universality of band gap descriptor and discovery of photovoltaic perovskites. *J. Am. Chem. Soc.*, 2024.

[6] Raisa-Ioana Biega, Marina R Filip, Linn Leppert, and Jeffrey B Neaton. Chemically localized resonant excitons in silver–pnictogen halide double perovskites. *J. Phys. Chem. Lett.*, 12(8):2057–2063, 2021.

[7] Maurizia Palummo, Eduardo Berrios, Daniele Varsano, and Giacomo Giorgi. Optical properties of lead-free double perovskites by ab initio excited-state methods. *ACS Energy Lett.*, 5(2):457–463, 2020.

[8] Julia Wiktor, Ursula Rothlisberger, and Alfredo Pasquarello. Predictive determination of band gaps of inorganic halide perovskites. *J. Phys. Chem. Lett.*, 8(22):5507–5512, 2017.

[9] Xin-Gang Zhao, Gustavo M Dalpian, Zhi Wang, and Alex Zunger. Polymorphous nature of cubic halide perovskites. *Phys. Rev. B*, 101(15):155137, 2020.
